# Perceptual Transparency From Cast Shadow

**DOI:** 10.1177/2041669519844272

**Published:** 2019-06-02

**Authors:** Takahiro Kawabe

**Affiliations:** NTT Communication Science Laboratories, Kanagawa, Japan

**Keywords:** cast shadow, transparency, surface perception, spatial frequency difference

## Abstract

This study examined how a shadow contributes to the perception of a transparent surface. As stimuli, we used computer graphics images in which a transparent surface with a color-mosaic pattern casts a shadow onto a background surface. We manipulated two parameters: (a) the spatial heterogeneity of the transmittance of the transparent surface and (b) the size of the light source shining on the transparent surface and its background. The latter parameter determined the blurriness of shadows. Observers judged whether the stimulus image contained a transparent surface or not. We found that the proportion of reports identifying a transparent surface was dependent on both parameters we tested. Specifically, a high spatial heterogeneity of transmittance decreased the proportion of reports of a transparent surface; this was possibly because globally defined X-junctions, which were one of the cues to perceptual transparency, perceptually broke down. On the other hand, blurred shadows were effective even when the global X-junctions were not effective. Locally defined X-junctions only moderately contributed to perceptual transparency. The results indicate that in addition to global and local X-junctions, blurred shadows are image features that elicit the perception of transparency from a cast shadow. A large individual difference as to which information each participant used as a cue to perceptual transparency was also discussed.

## Introduction

Human observers detect a transparent surface from various image cues (Sayim & Cavanagh, 2011). For example, when the edges of overlapped surfaces produce specific spatial patterns of luminance and chromaticity, so-called X-junctions, observers tend to perceive these surfaces overlapping with transparency (Adelson & Anandan, 1990; Beck & Ivry, 1988; Khang & Zaidi, 2002; Koenderink, van Doorn, Pont, & Wijntjes, 2010). In addition to luminance and chromaticity components, several image features such as motion (Adelson & Movshon, 1982; Qian, Andersen, & Adelson, 1994; Snowden, Treue, Erickson, & Andersen, 1991), binocular disparity (Akerstrom & Todd, 1988; Pollard & Frisby, 1990), dynamic image deformations (Kawabe, Maruya, & Nishida, 2015), and spatial frequency differences between overlapped surfaces (Fleming & Bülthoff, 2003; Kawabe & Miura, 2004, 2005; Kingdom & Keeble, 2000) promote the visual segregation of overlapping surfaces and eventually elicit perceptual transparency.

**Figure 1.**
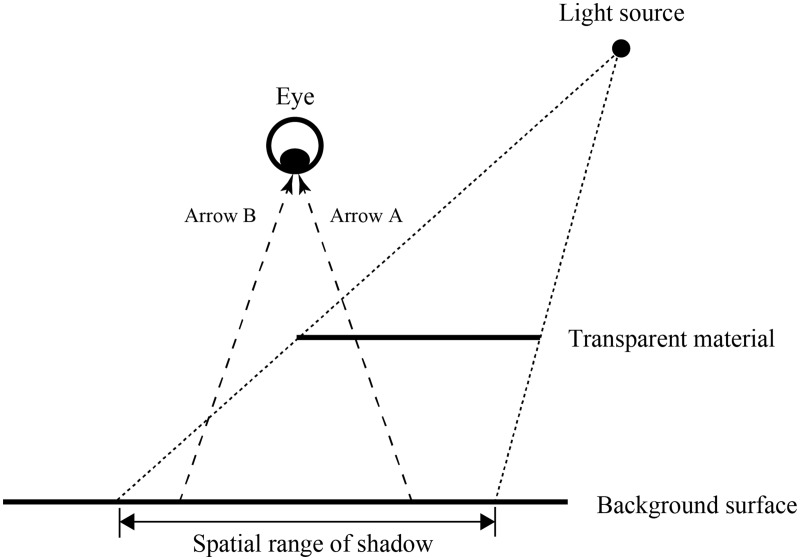
Schematic illustration of the way in which light that does or does not penetrate the body of a transparent material reaches an observer’s eye.

**Figure 2.**
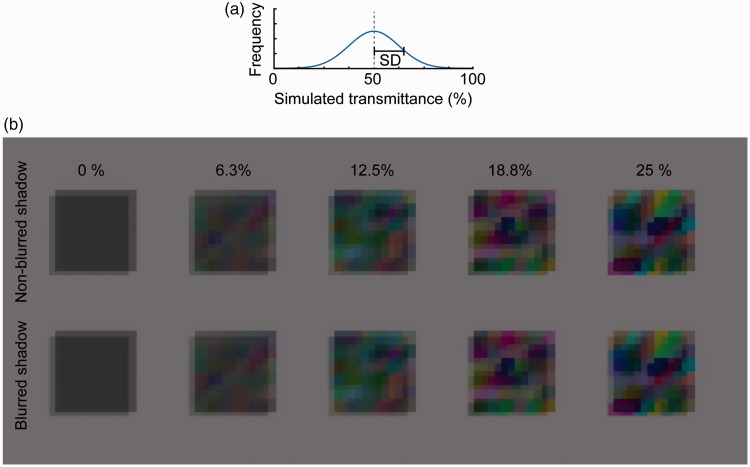
(a) A Gaussian function centered on 50% of simulated transmittance. The standard deviation (*SD*) of this function is manipulated. (b) Examples of stimuli as used in Experiment 1, wherein the spatial heterogeneity of transmittance (*SD*) and shadow blurriness were manipulated.

In this study, we focused on a specific physical situation that has not been closely examined, wherein a transparent object casts its shadow onto the surface of a background (Figure 1). Light that penetrates the body of the transparent material is reflected from the background surface. The spatial pattern of the reflected light is the shadow of the transparent material. Some portions of the image of the shadow again penetrate the body of the transparent material and reach the eyes of observers (Arrow A in Figure 1), while other portions directly reach the eyes of observers without such penetration (Arrow B in Figure 1). The shadow portion whose image performs the extra penetration is physically darker than the rest of the shadow. In retinal images, the difference in luminance across the shadow likely causes X-junctions, which are one of the strong triggers of perceptual transparency (see the contour junctions in Figure 2(a)). Unless other cues such as textures in the background surface are available, the X-junctions formed between the edge of a transparent surface and the edge of a shadow are the only cues that can trigger perceptual transparency.

However, it is unclear whether this simple explanation for perceptual transparency due to a cast shadow that includes X-junctions can be applied to a situation in which transmittance is not homogeneous across the surface of a transparent material (Figure 2(a)). Figure 2(b) shows some example images in which a transparent surface has spatially heterogeneous transmittance, and that surface casts its shadow on the background surface. As is clear from Figure 2(b), a shadow cast on the background has spatial luminance and chromaticity variations. Moreover, further modulations of luminance and chromaticity occur when the spatially heterogeneous shadow again penetrates the body of the transparent material with spatially heterogeneous transmittance. The right panel of Figure 2(b) shows that as the spatial homogeneity of transmittance increases, global X-junctions are less clear (here we mean global X-junctions that are formed globally between the edge of a transparent surface and the edge of a cast shadow, not the X-junctions that are formed locally by the edges of each cell of color-mosaic patterns.) Because perceptual transparency studies generally assume that the transmittance of a transparent material is spatially homogeneous, it is unclear whether the visual system will detect transparent materials when the materials have spatially heterogeneous transmittance. To address this issue, this study manipulated the spatial heterogeneity of transmittance across the surface of a transparent material.

In addition to the global X-junctions, we focused on the blurriness of a shadow made by a transparent material. The blur at the edge of a shadow is dependent on the size of the light source; a larger light source produces stronger blurriness at the edge of a shadow. As described earlier, the spatial frequency difference between foreground and background is a cue to perceptual transparency and translucency (Fleming & Bülthoff, 2003; Kawabe & Miura, 2004, 2005; Kingdom & Keeble, 2000). Therefore, it was possible that the blurriness of a shadow facilitates the segregation between a transparent surface and its shadow, so we also manipulated the size of the light source.

Accordingly, the purpose of this study was twofold. The first was to test whether the spatial heterogeneity of transmittances could affect perceptual transparency. The second was to check whether the blurriness of a shadow could promote perceptual transparency. In Experiment 1, we found that as the spatial heterogeneity of transmittance increased, a transparent material was reported less often. However, the blurred shadow still served as an effective cue to perceptual transparency even when the spatial heterogeneity of transmittance was at the top of the parameter range we manipulated. In Experiments 2 and 3, we confirmed that the blurred shadow caused perceptual transparency even when global X-junction cues were partially (Experiment 2) and completely (Experiment 3) eliminated from the stimuli. In the stimuli of Experiment 3, although global X-junctions were eliminated, local X-junctions formed between local patches on the surface and their shadows existed. The results showed that without the blurriness of shadow, the effect of the local X-junctions was only moderate. The results indicate that perceptual transparency elicited by a cast shadow generally comes from surface segregation on the basis of global and local X-junctions, and that a blurred shadow contributes to perceptual transparency even if the global and local X-junctions’ influence breaks down due to strong heterogeneity of transmittance in the transparent surface.

## Experiment 1

### Method

#### Observers

Twelve naive people (seven women and five men) participated in the experiment. Their mean age was 32.3 years (*SD* = 9.12). They reported having normal or corrected-to-normal visual acuity. They were recruited from outside the laboratory and received payment for their participation. Ethical approval for this study was obtained from the ethics committee at Nippon Telegraph and Telephone Corporation (Approval number: H28-008 by NTT Communication Science Laboratories Ethical Committee). The experiments were conducted according to principles that have their origin in the Helsinki Declaration. Written informed consent was obtained from all participants in this study.

#### Apparatus

Stimuli were presented on a 21-in. iMac (Apple Inc. USA) with a resolution of 2,048 × 1,152 pixels and a refresh rate of 60 Hz. The outputs of the monitor were gamma corrected. The CIE coordinates of the maximum intensity for each RGB channel were R (*x* = 0.6701, *y* = 0.3257, 45.2 cd/m^2^), G (*x* = 0.2597, *y* = 0.7042, 132.2 cd/m^2^), and B (*x* = 0.1483, *y* = 0.048, 11.1 cd/m^2^), which were measured using a colorimeter (Bm-5 A, Topcon, Japan). A computer (iMac, Apple Inc., USA) controlled stimulus presentation, and data were collected with PsychoPy v1.83 (Peirce, 2007, 2009).

#### Stimuli

**Figure 3.**
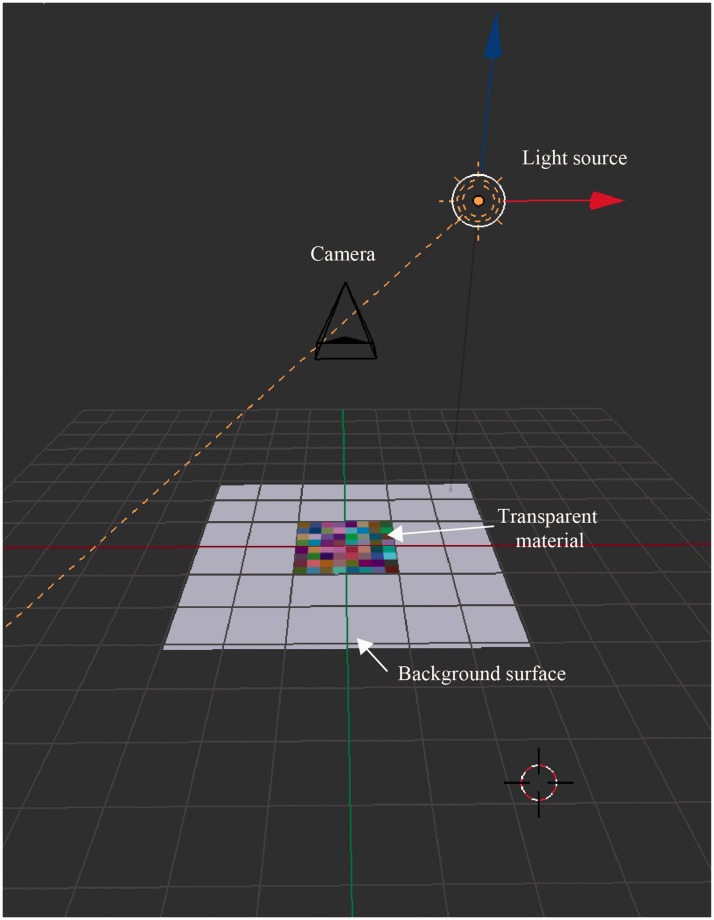
Geometry of a transparent material, a background surface, a camera, and a light source.

Stimuli were computer-rendered images that were created by using Blender (https://www.blender.org). Figure 3 shows the geometry of a background surface, a transparent material, a light source, and a camera. A diffuse gray material was used as the background surface, whose luminance was 76.6 cd/cm^2^ on the display. A transparent square surface, which subtended 4.6° × 4.6° of visual angle, had a 6 × 6 color mosaic pattern, each cell of which was given a unique transmittance value (see Figures 2 and 3). For each RGB channel, the transmittance value was randomly sampled from a Gaussian distribution that was centered on 50% transmittance. We selected the *SD* of the Gaussian distribution from the following nine levels: 0.0, 3.0, 6.3, 9.4, 12.5, 15.6, 18.8, 21.9, and 25.0%. With an *SD* of 0%, the transmittance of the transparent surface was spatially homogeneous. As the *SD* increased, the heterogeneity of transmittance increased. The simulated distance between a transparent material and background was 12.5 cm. The Sun, as a unitary light source of the scene, diagonally lit the scene from a top-right position and produced a shadow of the transparent surface at the bottom-left of the surface. We manipulated the size of the light sources between the following two levels: 0.001 and 0.1 (in blender, no unit is given to the size of light sources). The former and latter sizes of the light source produced shadows with sharp and blurred edges, respectively. We call the latter shadow with blurred edges as blurred shadow. For rendering stimulus images, Cycles render was used. Full global illumination was employed for a light path calculation.

#### Procedure

The observers sat approximately 64 cm from the display. Each observer individually performed a task in a separate chamber. For each trial, a stimulus image was presented at the center of the screen. The task of the observers was to report whether the stimulus image contained a transparent surface or not. The stimulus image was presented until the observers made their judgment. Each observer performed two sessions, each consisting of 180 trials (=2 levels of light source size × 9 levels of the *SD*s of transmittance × 10 repetitions). The order of the stimulus images was pseudorandomized across the observers.

### Results

**Figure 4.**
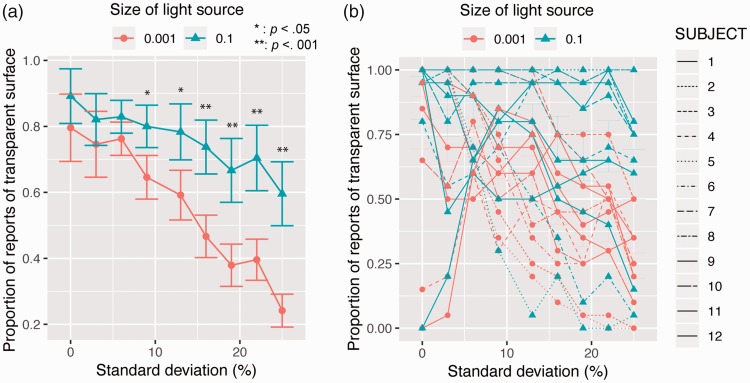
Experiment 1 results. (a) The proportion of reports of a transparent surface is plotted as a function of the *SD* of transmittance. Asterisks show the *SD* conditions that produced significant differences between the two light source size conditions, and the number of asterisks denotes significance levels. Error bars show standard errors of mean (*N* = 12). (b) Individual proportions are shown.

For each condition, the proportion of reports of a transparent surface was calculated for each individual. Figure 4(a) shows the averaged proportions as a function of the *SD* of transmittance. Using the proportions, we conducted a repeated two-way analysis of variance with the light source size and the *SD* of transmittance as within-subject factors. The main effect of the light source size was significant, *F*(1, 11) = 12.150, *p* = .0051, *partial η*^2 ^= 0.52. The main effect of the *SD* of transmittance was also significant, *F*(8, 88) = 7.148, *p* < .0001, *partial η*^2 ^= 0.39. Interaction between the two factors was also significant, *F*(8, 88) = 5.029, *p* < .0001, *partial η*^2 ^= 0.31. As the asterisks in Figure 4 show, the simple main effect of the light source size on the basis of the significant interaction was significant when the *SD*s of transmittance were 9.4 and 12.5 (*p* < .01), and when they were 15.6, 18.8, 21.9, and 25.0% (*p* < .001). The simple main effect of the *SD* of transmittance was significant when the light source size was 0.001 (*F* = 11.152, *p* < .0001) and 0.1 (*F* = 2.425, *p* = .0162). Multiple comparison tests of the simple main effect of the *SD* of transmittance showed that when the light source size was 0.001, the proportion in the 0 *SD* condition was significantly different from the conditions with the *SD* more than 15.6°. However, when the light source size was 0.1, no significant difference was observed between any pair of *SD* conditions (*p* > .05) except a pair, the *SD* 0 and *SD* 25.0 (*p* < .05).

### Discussion

The results showed that the proportion of reports of a transparent surface was dependent on both the *SD* of transmittance and the size of the light source. With regard to the *SD* of transmittance, as the *SD* increased, the reports of a transparent surface decreased. The results are consistent with the idea that when the spatial heterogeneity of transmittance increases, global X-junctions formed between the edge of a transparent surface and the edge of its shadow are not perceptually evident, and hence the stimulus image lacks a strong cue to trigger perceptual transparency. On the other hand, even when the *SD*s of transmittance were high, the proportion of reports for a transparent surface remained high when the size of the light source was 0.1. The results indicate that a blurred shadow served as an effective cue to trigger perceptual transparency. It is possible to consider that the condition with an *SD* of 0 can be regarded as a baseline because the stimulus configuration is akin to stimuli as used in studies on conventional perceptual transparency (Adelson & Anandan, 1990; Beck & Ivry, 1988). In our data, when the shadow was blurred, the performance under the 0 *SD* condition was not significantly different from the performance under the condition where the *SD*s were less than 21.9. On the other hand, when the shadow was not blurred, the performance under the 0 *SD* condition was not significantly different from the performance under the condition where the *SD*s were less than 15.6°. The results suggest that the blurriness of cast shadows serves as an additional cue causing perceptual transparency, which was comparable to conventional transparency based on X-junctions.

We also observed large individual differences for the effect of the blurred shadow on the proportion of reports of a transparent surface. As shown in Figure 4(b), in half of the observers, the blurred shadow was an effective cue to perceptual transparency when the *SD* of transmittance was high. However, among the other half of observers, reports of a transparent surface decreased with the *SD* of transmittance even when the size of the light source was 0.1. We suppose that the latter group of observers might rely solely on the presence or absence of global X-junctions in performing this task. They might report “no transparency” when global X-junctions were not perceptually evident, even when a blurred shadow cue was available.

**Figure 5.**
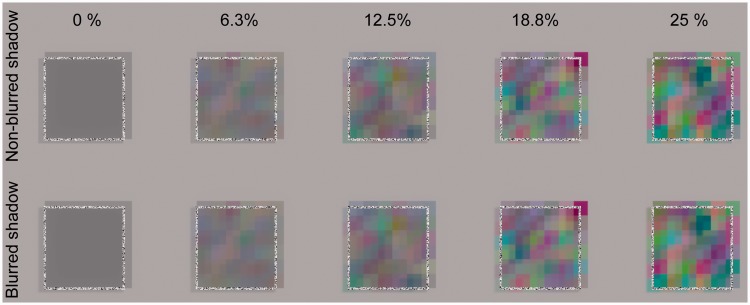
Some example images of stimuli as used in Experiment 2, wherein the X-junctions in the stimuli were masked by a white-noise occluder. Please enlarge the figure to see the difference between the blurred and nonblurred shadow conditions.

In the next experiment, we investigated how the attenuation of the global X-junctions influenced perceptual transparency from cast shadow. In Experiment 2, an occluder was added to the stimuli so that the global X-junctions formed between the edge of a transparent surface and the edge of its shadow were not perceptually evident (Figure 5), consistent with a previous study (Kasrai & Kingdom, 2002) showing that the occlusion of the X-junction reduced perceptual transparency. We again asked the observers to report whether the stimulus image contained a transparent surface or not. In this task, we expected that it was difficult (Experiment 2) for the observers to use global X-junctions as a cue. Hence, it was expected that we could attenuate the effect of the global X-junctions on the judgment of perceptual transparency.

## Experiment 2

### Method

#### Observers

Eleven naive people (nine women and two men) participated in the experiment. Three of them had participated in Experiment 1 while they were still unaware of the purpose of the experiment. Their mean age was 35.5 years (*SD* = 10.4). They reported having normal or corrected-to-normal visual acuity. They were recruited from outside the laboratory and received payment for their participation.

#### Apparatus

Identical to that used in Experiment 1.

#### Stimuli

Stimuli were basically identical to those used in Experiment 1 except for the following. The X-junctions in the stimulus images as used in Experiment 1 were covered with a rectangular frame as an occluder (Figure 5). The thickness of the frame was 0.12°. The frame was filled with white noise, each cell subtending 0.04° × 0.04°.

#### Procedure

The procedure was identical to that used in Experiment 1 except for the following. The observers were asked to ignore the occluder and judge whether the stimulus image contained a transparent surface or not.

### Results

**Figure 6.**
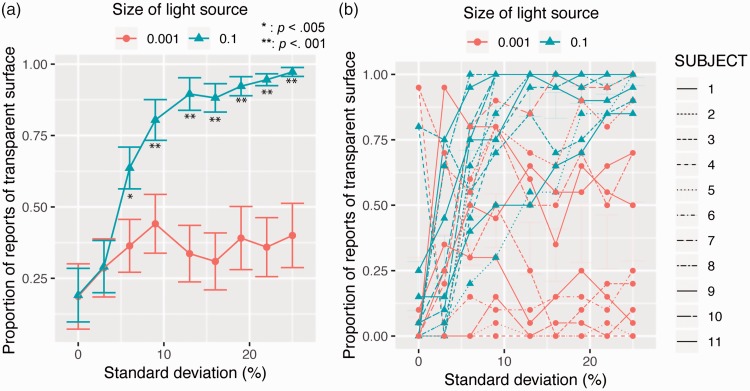
Experiment 2 results. (a) Averaged proportion of reports of a transparent surface is plotted as a function of the *SD* of transmittance. Error bars denote standard error of mean (*N* = 11). (b) Individual proportions. Each line shows an individual proportion.

As in Experiment 1, for each condition, the proportion of reports of a transparent surface was calculated for each individual. Figure 6(a) shows the averaged proportions as a function of the *SD* of transmittance. Using the proportions, we conducted a repeated two-way analysis of variance with the light source size and the *SD* of transmittance as within-subject factors. The main effect of the light source size was significant, *F*(1, 10) = 27.518, *p* = .0004, partial *η*^2 ^= 0.73. The main effect of the *SD* of transmittance was also significant, *F*(8, 80) = 9.225, *p* < .0001, partial *η*^2 ^= 0.48. Interaction between the two factors was also significant, *F*(8, 80) = 19.076, *p* < .0001, partial *η*^2 ^= 0.66. As the asterisks in Figure 6(a) show, the simple main effect of the light source size was significant when the *SD*s of transmittance were 6.3 (*p* < .005), and also when they were 9.4, 12.5, 15.6, 18.8, 21.9, and 25.0% (*p* < .001). The simple main effect of the *SD* of transmittance was significant when the light source size was 0.1 (*F* = 20.713, *p* < .0001) but not when it was 0.1 (*F* = 1.342, *p* < .23).

### Discussion

In this experiment, we tried to attenuate the effect of the global X-junctions by adding an occluder and found that the proportion of perceptual transparency reports increased when the *SD* of transmittance was large but only when the light source size was 0.1. That is, without the global X-junctions, perceptual transparency occurred only when the transparent surface was spatially heterogeneous and the image included the shadow with blurred edges. The results again support our idea that the blurriness of a shadow facilitates the visual segregation between a transparent surface and its shadow.

We also observed two sorts of individual differences in this experiment. First, when the transmittance was spatially homogeneous, two of the observers frequently reported a transparent surface. This may be because these two observers used the luminance relationship between the overlapped and nonoverlapped areas as a cue to perceptual transparency. Second, some observers frequently reported a transparent surface when the surface with spatially heterogeneous transmittance casts a shadow with clear edges. This is possibly because these observers used, as a cue to perceptual transparency, conventional X-junctions that were locally made between surface mosaics and cast shadows.

On the other hand, an important point is that as shown in Figure 6(b), when the surface with spatially heterogeneous apparently dropped a shadow with blurred edges, all observers robustly reported a transparent surface. We thus conclude that observers will reliably use the blurriness of shadow edges as a cue to perceptual transparency, though other cues such as the luminance relationship between overlapped and nonoverlapped areas and local X-junctions are also used when blurred shadow edges are not available.

**Figure 7.**
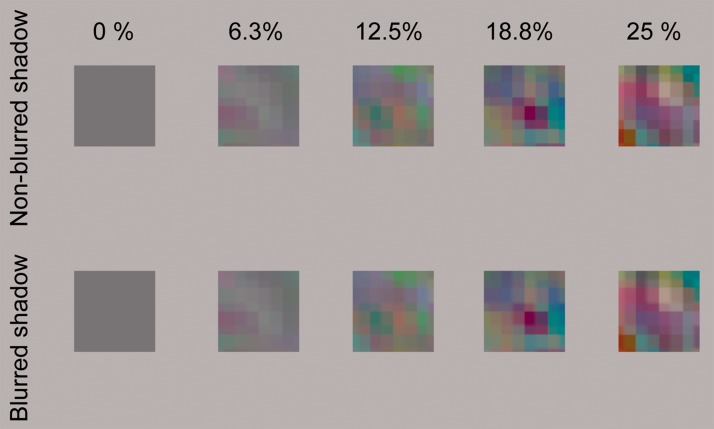
Some example images of the stimuli used in Experiment 3 in which nonoverlapped areas of transparent surfaces and their shadows were eliminated. Please enlarge the figure to see the difference between the blurred and nonblurred shadow conditions.

In the next experiment, we further investigated the relationship between the local X-junctions and the shadow blurriness. In Experiment 3, we trimmed the stimulus image so that it contained only the areas in which a transparent surface and its cast shadow were completely overlapped (Figure 7). We again asked the observers to report whether the stimulus image contained a transparent surface or not. In this task, it was impossible (Experiment 3) for the observers to use global X-junctions as a cue, so they could only use other information as a cue to transparency. Hence, it was expected that we could eliminate the effect of the global X-junctions on the judgment of perceptual transparency. In the stimuli of Experiment 3, however, the local X-junctions were still available. Hence, the purpose of Experiment 3 was to check how strongly X-junctions influenced the perception of transparent surfaces from cast shadow.

## Experiment 3

### Method

#### Observers

The same 11 naive people as in Experiment 2 participated in this experiment. They were still unaware of the purpose of the experiment.

#### Apparatus

Identical to that used in Experiments 1 and 2.

#### Stimuli

Stimuli were basically identical to those used in Experiment 1 except for the following. The stimulus images as used in Experiment 1 were trimmed so that the stimulus image in this experiment contained areas wherein a transparent surface and its cast shadow overlapped completely. As a result of the trimming, each stimulus image contained a 2.6 × 2.6 square area at its center (Figure 7).

#### Procedure

The procedure was identical to that used in Experiment 1.

### Results

**Figure 8.**
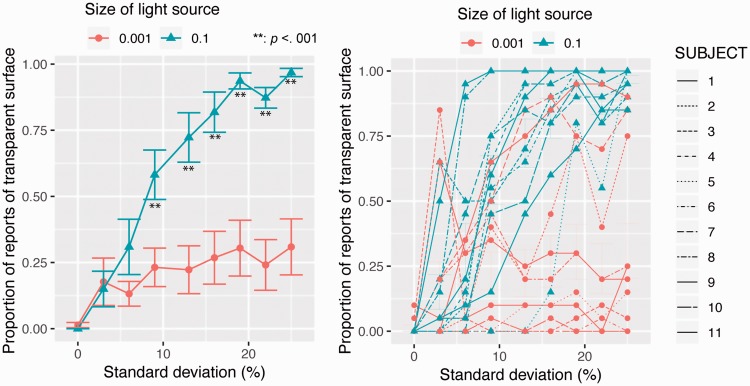
Experiment 3 results. (a) The averaged proportion of reports of a transparent surface is plotted as a function of the *SD* of transmittance. Error bars denote standard error of the mean (*N* = 11). (b) Individual proportions. Each line shows an individual proportion.

As in Experiment 2, for each condition, the proportion of reports of a transparent surface was calculated for each individual. Figure 8(a) shows the averaged proportions as a function of the *SD* of transmittance. Using the proportions, we conducted a repeated two-way analysis of variance with the light source size and the *SD* of transmittance as within-subject factors. The main effect of the light source size was significant, *F*(1, 10) = 25.295, *p* = .0005, partial *η*^2 ^= 0.72. The main effect of the *SD* of transmittance was also significant, *F*(8, 80) = 26.514, *p* < .0001, partial *η*^2 ^= 073. Interaction between the two factors was also significant, *F*(8, 80) = 16.287, *p* < .0001, partial *η*^2 ^= 0.61. As the asterisks in Figure 8(a) show, the simple main effect of the light source size was significant when the *SD*s of transmittance were 9.4, 12.5, 15.6, 18.8, 21.9, and 25.0% (*p* < .001). The simple main effect of the *SD* of transmittance was significant when the light source size was 0.001 (*F* = 2.846, *p* < .01) and 0.1 (*F* = 20.713, *p* < .0001).

### Discussion

When X-junctions were eliminated from the stimulus images, no reliable cues other than a blurred shadow existed. Hence, as we expected, the proportion of transparent surface reports was high when the *SD* of transmittance was high. The results again support our idea that the blurriness of a shadow facilitates making the segregation between a transparent surface and its shadow.

In comparison with the results of Experiment 2, individual differences were relatively low when the spatial heterogeneity of transmittance was low (that is, the *SD* of the transmittance was low). This was because the luminance relationship between the overlapped and nonoverlapped areas was no longer available as a cue to perceptual transparency. On the other hand, some observers frequently reported transparent surfaces even when the surface with spatially heterogeneous transmittance cast a shadow with clear edges. As in Experiment 2, these observers might use the locally generated X-junctions as a cue to perceptual transparency. Importantly, however, when the surface with spatially heterogeneous transmittance casts a shadow with blurred edges, all observers consistently reported a transparent surface, which again indicates that the blurriness of cast shadow edges is a robust cue to perceptual transparency from a cast shadow.

## Conclusions

We report that perceptual transparency resulting from cast shadows stemmed from two variable image features. The first was the X-junctions formed between the edge of a transparent surface and the edge of its cast shadow. The second was the blurred shadow generated by an extended light source.

**Figure 9.**
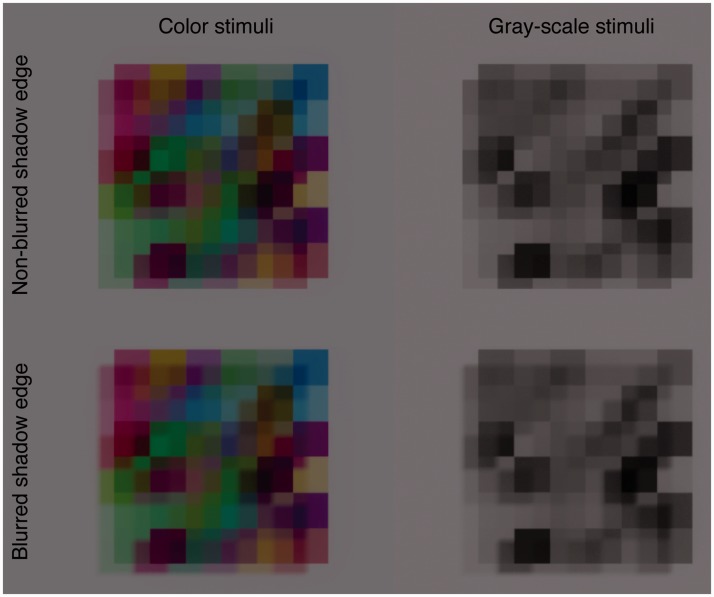
Comparison of the shadow edge blurriness between color and gray-scale stimuli. In both stimuli, the impression of perceptual transparency is stronger with than without blurred shadow edges.

This is the first study to show that global X-junctions break down as a cue when high spatial heterogeneity of transmittance is given to a transparent surface. As the *SD*s of transmittance were high, the resultant image intensity of each patch of the color mosaic patterns was broadly distributed. Hence, in order to detect global X-junctions in this scenario, it was necessary for the observers to group (or average) the several patches with different intensities and separate the signals that originated in the edge of a transparent surface from the signals that originated in the edge of a shadow. It is natural to assume that such grouping and processing are more difficult when dealing with higher *SD*s of transmittance. Still, the precise luminance distribution that hampers the detection of global X-junctions is unclear and is a topic for investigation in future studies. Because the effect of shadow blurriness on perceptual transparency would probably be the same in gray-scale stimuli (Figure 9), this appears to be a promising way to use monochrome stimuli to investigate what kind of luminance distribution disassociates perceptual transparency from the global X-junctions.

The effect of a blurred shadow on perceptual transparency may be related to earlier evidence that a spatial frequency difference between two overlapping surfaces is an effective cue to perceptually segregate them from each other. In the two-dimensional space of our stimulus image, a blurred shadow was spatially offset from a transparent surface. That is, low spatial frequency components of a blurred shadow were spatially offset from high (and low) spatial frequency components of a transparent surface. Previous studies have reported that the spatial offset between low and high spatial frequency components helped observers to distinguish the components from each other. For example, Kingdom and Keeble (2000) showed that when two orientation-modulated gratings are superimposed in antiphase, thresholds to segregate the gratings decreased with the increase of the difference in luminance spatial frequency between the gratings. Kawabe and Miura (2004, 2005) showed that the effect of spatial frequency difference on perceived transparency depended on the spatial configurations of overlapped surfaces.

A different line of research has reported that the phase difference between low and high spatial frequency components contributes to perceptual transparency. For example, Morrone and Burr (1988) showed that a phase difference between low and high spatial frequency components caused a transparent surface segregation between the components. The spatial offset of the edges in this study may involve the phase-based transparency that Morrone and Burr reported. In addition, Fleming and Bülthoff (2005) showed that the polarity reversal of high spatial frequency components in an image of a cube caused the impression of translucency of the cube. Thus, the phase inconsistency as a strong cue to perceptual transparency might be involved with the perceptual transparency resulting from a blurred shadow. It is worth checking how dependent the perceptual transparency from a blurred shadow is on the size of the spatial offset between a transparent surface and a blurred shadow in future studies. Finally, it has been also proposed that image blur itself is a cue to perceptual translucency even when the foreground translucent surface has no spatial pattern (Singh & Anderson, 2002). It is also an intriguing issue how spatial patterns on a foreground transparent surface interact with blurred background patterns in judging the translucency of the foreground surface.
